# Cancer Stem Cells

**DOI:** 10.1155/2011/269437

**Published:** 2011-04-10

**Authors:** Bo Lu, Shih-Hwa Chiou, Eric Deutsch, Aurelio Lorico

**Affiliations:** ^1^Department of Radiation Oncology, Vanderbilt-Ingram Cancer Center, Vanderbilt University School of Medicine, Nashville, TN 37232, USA; ^2^Institute of Pharmacology/Clinical Medicine, National Yang-Ming University, Taipei 112, Taiwan; ^3^Département de Radiothérapie, Institut Gustave Roussy, rue Camille Desmoulins, Villejuif, France; ^4^Department of Drug Development, Nevada Cancer Institute, Las Vegas, NV 89135, USA

The concept of cancer stem cells has generated significant interests among cancer researchers. This has led to proliferative number of publications on the subject in the recent years and eventually controversies because of conflicting data in the literature. As we try to find relevance of the stem cell concept to various aspects of cancer biology, we also discover individual differences that drive the cancer stemness. Nevertheless, the concept of cancer stem cell is likely to stay and is already making huge impact as we develop novel diagnostics and therapeutics for cancer.

In this special issue, we selected reviews on the unique stem cell biology in breast cancer, head and neck cancer, lung cancer, and brain malignancy and touched the topic of therapeutic resistance as a consequence of cancer stem cells. Individual research papers were also selected to demonstrate the relevance of cancer stem cells to cancer metastasis and apoptosis.

As we embrace the concept of cancer stem cells, it is important to realize its limitations. We have learnt that antiangiogenic drugs that attack tumor blood vessels, an important aspect of cancer biology, have not been able to cure cancer and may cause unique side effects in patients. By the same token, toxicities are of concern when the stem cells of normal tissues are injured by agents that target stem cell pathways. With this issue, we aim to provoke discussion and awareness of cancer stem cell biology, as some of research findings are being translated and making their way to our oncology practice.

*Bo Lu* *Bo Lu* *Shih-Hwa Chiou* *Shih-Hwa Chiou* *Eric Deutsch* *Eric Deutsch* *Aurelio Lorico* *Aurelio Lorico*

